# Lifestyle modifications after the diagnosis of gynecological cancer

**DOI:** 10.1186/s12905-021-01391-5

**Published:** 2021-06-28

**Authors:** Daniela Paepke, Clea Wiedeck, Alexander Hapfelmeier, Marion Kiechle, Christine Brambs

**Affiliations:** 1 Department of Gynecology and Obstetrics, Technical University of Munich, Munich, Germany; 2 Institute of Medical Informatics, Statistics and Epidemiology, School of Medicine, Technical University of Munich, Munich, Germany; 3 Institute of General Practice and Health Services Research, School of Medicine, Technical University of Munich, Munich, Germany

**Keywords:** Lifestyle, Gynecological cancer, Nutrition, Physical activity, Alcohol consumption, Nicotine consumption, Stress

## Abstract

**Background:**

The influence of lifestyle factors on the quality of life, incidence and tumor recurrence has been evaluated in several studies and is gaining increasing importance in cancer research. However, the extent of the influence of such lifestyle factors on the quality of life of cancer patients remains largely unclear, as does the number of patients actually pursuing these lifestyle changes. The purpose of this study was to examine the prevalence and predictors of lifestyle changes in patients with gynecological cancer.

**Methods:**

The survey consisted of a pseudonymous questionnaire that was conducted from January to May 2014 via a telephone interview with 141 patients with a gynaecological malignancy who had undergone surgery at our Department of Gynaecology and Obstetrics. Lifestyle factors (diet, physical activity, stress level, alcohol and nicotine consumption) prior to and after the diagnosis of cancer were evaluated.

**Results:**

89% (n = 125) of the patients reported lifestyle changes after being diagnosed with cancer. There was a significant association between the implementation of lifestyle changes and age as well as the use of complementary medicine. *Nutrition*: 66% of the patients (n = 93) consumed more fruit and vegetables and 65% ate less meat (n = 92). *Physical activity*: 37% (n = 52) reported no change in their exercise routine, 36% (n = 51) described a decrease, 27% (n = 38) an increase in their physical activity. *Subjective feeling of stress*: 77% of the patients (n = 108) described a reduction in their perceived level of stress. *Nicotine consumption*: 63% (n = 12) of the 19 patients who were smokers at the time of the diagnosis quit or reduced smoking thereafter. *Alcohol consumption*: 47% (n = 61/129) of the patients reduced their alcohol consumption.

**Conclusions:**

Most of the patients from our study group implemented lifestyle changes after being diagnosed with cancer. Prospective randomized trials are needed in order to determine the benefit of lifestyle changes (physical activity, dietary habits and stress reduction) for cancer survivors. The potential impact of lifestyle on the quality of life and the trajectory of the disease should be discussed with all oncological patients.

**Supplementary Information:**

The online version contains supplementary material available at 10.1186/s12905-021-01391-5.

## Background

There is an increasing body of literature regarding the influence of lifestyle factors such as nutrition, the consumption of nicotine and alcohol as well as exercise on the development of different types of tumors and lifestyle is playing an increasing role in the prevention of malignancies.

The impact of such lifestyle factors on the long-term survival and quality of life of cancer patients remains largely unclear, as does the question how many cancer patients actually implement lifestyle changes after the diagnosis of a malignancy. Some data suggest that 58% of long-term cancer survivors are overweight, 25% continue to smoke, 50% exercise and less than 20% report a sufficient fruit and vegetable consumption [1]. In the US only 20% of cancer patients implement the recommended 2.5 h of physical activity per week, and only 35% are not overweight [2].

Based on the existing body of evidence, lifestyle modifications affecting diet, body weight and physical activity might improve the prognosis of malignancies.

Nagle et al. and Dolecek et al. reported a prolonged survival in patients with ovarian cancer with an increased intake of fruit and vegetables while a higher consumption of meat and dairy products had a negative impact on survival in the same patient population [3, 4]. Contrary to the results published by Dolecek et al., the “Women ‘s Health Initiative Observational Study “ did not show an association between the consumption of certain food groups and an improved survival. This long-term national health study evaluated 161,808 postmenopausal women between 1995 and 2012 with the goal to establish strategies to help prevent cardiovascular disease, breast and colorectal cancer as well as osteoporotic fractures. The questionnaire (“Healthy Eating Index”) was used to assess the dietary routine during the trial period and to evaluate a potential association with mortality. Within the questionnaire the consumption of 12 dietary elements (total fruits including juice, whole fruits excluding juice, total vegetables, dark green and orange vegetables and legumes, total grains, whole grains, milk, meat and beans, oils, saturated fat, sodium, calories from solid fats/ alcoholic beverages/ added sugar) was assessed.

636 participants of the trial were diagnosed with ovarian cancer during the period of observation. There was no statistically significant impact of the consumption of different food groups prior to the diagnosis of ovarian cancer and the overall mortality in this subset of patients. However, a higher dietary quality according to Healthy Eating Index (2005) was associated with a significantly lower mortality, suggesting an influence of the overall nutrition on the course of the disease rather than different dietary components [5].

In the setting of an increasing number of overweight patients, an evaluation of a potential association between the body mass index (BMI) and the prognosis of gynecological malignancies is of great interest. The largest data collection on a potential influence of obesity on the survival of ovarian cancer patients was published by the Ovarian Cancer Association Consortium (OCAC) in 2015, including 21 trials and a total of 12,390 women. Women who had been obese (BMI ≥ 30) for one to five years prior to the diagnosis of ovarian cancer were shown to have a 12% increase in mortality [6]. Similarly, obesity (BMI > 25) was associated with a higher overall mortality in endometrial cancer patients [7, 8]. This difference in survival may be explained by different hormonal mechanisms as well as an insufficient dosing of chemotherapy in overweight patients [9].

The American Cancer Society recommends regular physical activity (at least 150 min per week and including weight training on at least two days) and a quick resumption of regular daily activities to cancer survivors [10].

The first prospective trial evaluating the influence of physical activity in 600 ovarian cancer patients with a median follow-up of 10.9 years was published in 2014. It reported a reduction of the cancer-specific and overall mortality by 26% and 24% respectively in women who reported regular vigorous physical activity before the cancer diagnosis [11].

The lifestyle intervention study LIBRE-1 (Lifestyle Intervention Study in Women with Hereditary Breast and Ovarian Cancer), a randomized, prospective trial aiming to test the feasibility of lifestyle modifications in BRCA-1 and -2 mutation carriers, showed that there was a significantly lower prevalence of cancer in participants who had been physically active during their adolescence (*p* = 0.019). Patients who were smokers prior to the diagnosis of cancer also showed a significantly higher prevalence of malignancy than non-smokers (*p* < 0.001). In the 68 patients evaluated as part of this study, non-diseased mutation carriers revealed a significantly higher physical activity level than diseased mutation carriers (*p* = 0.046) and diseased mutation carriers (22.5 ± 5 kg/m^2^) had a lower BMI compared to non-diseased mutation carriers (25 ± 8 kg/m^2^), however this difference did not reach statistical significance (*p* = 0.079) [12].

Studies have shown the negative effect of stress on multiple female conditions such as infertility and endometriosis [13, 14]. Previous studies have described a higher level of stress and depression in cancer patients which can lead to a reduction of quality of life, thus having insight into the emotional status of the patients is critical [15–17].

Davis et al. [18] evaluated spiritual growth as a potential area of posttraumatic growth in 241 ovarian cancer patients prior to surgery and one year post-operatively. Spiritual growth was measured by examining the three following items: meaning (e.g., “I have a reason for living”), peace (e.g., “I feel a sense of harmony within myself”), and faith (e.g., “I find comfort in my faith or spiritual beliefs”). An increase in peace was associated with lower rates of depression (*p* ≤ 0.001) and anxiety (*p* = 0.004) at one year. There was no statistically significant association between the changes in meaning and faith and rates of depression and anxiety. Changes in peace helped neutralize the effect of stressful life events on depression (*p* = 0.027) and anxiety (*p* = 0.05), resulting in the worst psychological outcomes after one year in patients with a high number of life events and a decrease in peace. These findings suggest that the quality of peace may be the most adaptive parameter of spiritual growth in cancer patients. Furthermore, changes in peace appear to reduce the effect of life events on the psychological well-being.

Furthermore, it should also be highlighted that patients undergoing surgery for gynecological malignancies can strongly benefit from specialized pre-/intra and post-operative care. The ERAS-protocol (“Enhanced Recovery After Surgery”) includes over 20 items such as adequate nutrition, early post-operative mobilization and pain management. Through implementation of this protocol, it was possible to significantly reduce the time of hospitalization, post-operative complications and a reduction of the use of opioids for pain management [20].

While including lifestyle modification strategies into oncological patient care is becoming more and more common, there is limited evidence on it. The goal of the current study was to add further data on the important aspect of lifestyle modifications in this group of patients in order to integrate relevant aspects into oncological treatment plans in the future.

## Materials and methods

From January to May 2014 we gathered data at the Department of Gynaecology and Obstetrics, Technical University of Munich (TUM), Germany using a structured pseudonymous questionnaire which was carried out via a telephone interview or e-mail.

### Study population

The telephone interview was conducted with women who had undergone gynaecological cancer surgery from January 2011 to December 2013 at the Department of Gynaecology and Obstetrics, Technical University Munich, Germany. In the beginning of the interview consent to participate in the study was obtained verbally after explaining the purpose of the study and its pseudonymous nature.

Inclusion criteria were age ≥ 18 years, command of the German language and the capacity to understand/respond to the questionnaire, i.e. no apparent dementia/ cognitive impairment or speech impairment.

Patients with benign- and borderline tumors were excluded from the study.

### Questionnaire

The survey questionnaire included 111 items and was developed in the German language. It included the state of disease (metastases, recurrence, oncological treatment), sociodemographic factors (age, education, marital status, employment, BMI), personal opinions regarding complementary and alternative medicine (CAM) and Health behaviour (lifestyle factors, nutritional habits, physical activity) (Additional file 1).

The questionnaire was drawn up by specialists in gynecology and obstetrics with additional qualifications in nutritional medicine and integrative oncology.

To develop the Questionnaire no previous source was used. The questions were selected in accordance with the recommendations of the German Nutrition Society and the German Cancer Society. After the questionnaire was created, it was discussed with oncology nurses and any questions that could be misunderstood were changed. The questionnaire was then validated on 10 patients and adjusted again.

The majority of questions were designed in the format of multiple-choice. To examine changes in the level of stress, patients were asked to compare the level of stress prior to and after the diagnosis of cancer, and to comment on potential changes. In addition to the self-assessment of the level of stress, an ordinal scale of the stress level pre- and post-diagnosis allowed a concrete comparison. This scale ranged from 1 to 10, correlating with the level of stress.

This paper focuses on the results of lifestyle changes while the prevalence of the use of CAM will be discussed in a separate publication.

### Statistical analysis

The distribution of quantitative date was described via mean ± standard deviation.

Absolute and relative frequencies were used to present qualitative data. T-tests and chi-squared tests was used to detect associations between sociodemographic characteristics and health behaviours. Our approach towards the data analysis was exploratory, without a specific a-priori hypothesis to prove and therefore without formal sample size calculation based on a power analysis of a statistical hypothesis test. The sample size of n = 141 is however sufficient for any of the performed analyses. Hypothesis testing was therefore conducted on exploratory two-sided 5% significance levels. Data management and statistical analyses were performed using excel and the statistical software IBM SPSS Statistics, Version 22 (IBM Corp., Armonk, N.Y., USA).

## Results

**Fig. 1:**
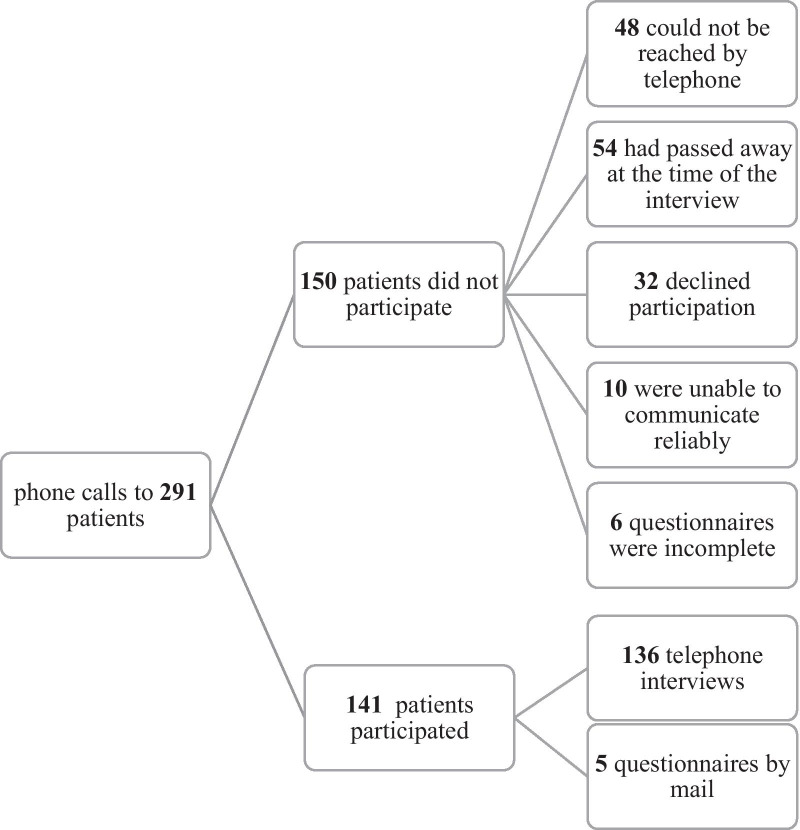
Flow-chart of participation and non-participation in the study

141 of 291 patients completed the questionnaire, corresponding to a participation rate of 48%. After exclusion of the patients that had passed away at the time of the interview, the participation rate amounts to 59%. Figure 1 depicts the participation in the study with inclusion of the reasons for non-participation.

The majority of patients suffered from ovarian cancer (64%, n = 90). 23% (n = 32) were suffering from endometrial cancer, 9% (n = 13) from cervical cancer and further 4% (n = 6) from vulvar cancer.

### Subjective lifestyle evaluation prior to the cancer diagnosis

Patients were initially asked to assess their lifestyle prior to the diagnosis of cancer (“Did you lead a healthy life prior to the diagnosis of cancer?”). 105 patients described their lifestyle as healthy, 12 patients as average and 23 as not healthy (Table 1). Patients claiming to have pursued a healthy lifestyle were evaluated regarding obesity, nutrition and physical activity. 54% (n = 57) were found to have a BMI ≥ 25 and were considered overweight. 26% (n = 27) consumed meat daily or several times per day, and 70% (n = 73) ate only one to two portions of fruits or vegetables per day. In 26% of the patients (n = 27), there was an obvious lack of activity (physical exercise less than one to two times per week).

**Table 1:** Self-assessment of lifestyle prior to malignancy

Lifestyle prior to diagnosis of malignancy	Amount of patients (total n = 140)	%
„Healthy“	105	75
„Not healthy“	23	16
„Average“	12	9

89% (n = 125) of the patients reported positive lifestyle changes of at least one of the factors evaluated (smoking, alcohol consumption, nutrition, physical activity, stress level).

Patients who modified their lifestyle positively were significantly younger than those who did not (58.4 versus 65.4 years; *p* = 0.03, Table 2).

**Table 2:** Lifestyle changes in relation to several variables

Characteristics	Lifestyle changes since the diagnosis of cancer		
	Healthier	Unchanged/ less healthy	*p* value
Age ± SD	58.4 ± 12.0	65.4 ± 8.7	*p* = 0.03
Lifestyle assessment prior to the malignancy			
„Healthy“ (n = 105)	92 (88%)	13 (12%)	*p* = 0.5
„Not healthy“ (n = 23)	22 (96%)	1 (4%)	
BMI			
Not overweight (n = 65)	60 (92%)	5 (8%)	*p* = 0.2
Overweight (n = 71)	60 (85%)	11 (15%)	
CAM use			*p* = 0.01
Use of CAM (n = 84)	79 (94%)	5 (6%)	
No use of CAM (n = 57)	46 (81%)	11 (19%)	
Highest level of education			*p* = 0.7
No school diploma/ primary school + 4 years (n = 37)	32 (87%)	5 (13%)	
Middle school diploma (n = 45)	39 (87%)	5 (13%)	
High school diploma (n = 59)	54 (91%)	5 (9%)	

A lifestyle improvement was detected in 96% (n = 22) of the patients who described their own lifestyle as unhealthy prior to the diagnosis of cancer. 88% (n = 92) of those who claimed to have a healthy lifestyle prior to the cancer diagnosis were able to further improve their lifestyle (*p* = 0.5, Table 2).

Patients who used CAM treatments were significantly more likely to implement lifestyle improvements (*p* = 0.01, Table 2). Lower Body-Mass-Index (*p* = 0.2, Table 2) and a higher education level (*p* = 0.7, Table 2) showed a positive relation to lifestyle modifications.

### Modification of individual lifestyle factors

#### Tobacco consumption

A reduction in tobacco consumption was found in 63% (n = 12) of current and former smokers after the cancer diagnosis. Four patients reduced their tobacco intake and eight patients quit smoking completely.

#### Alcohol consumption

91% (n = 129) of the patients regularly consumed alcohol prior to the cancer diagnosis. 84% (n = 109) drank alcohol up to three times per week and 16% (n = 20) drank alcohol daily. Overall, 47% (n = 61) reduced the consumption of alcohol after the diagnosis of a malignancy.

A similar reduction of alcohol intake was noted in patients with average alcohol consumption (up to 3x/week) and in patients with daily alcohol intake (*p* = 0.8, Table 3).

**Table 3:** Changes in alcohol consumption

Alcohol consumption prior to cancer diagnosis	Changes in alcohol consumption after cancer diagnosis		
	Reduction	Unchanged/increase	*p* value
Up to 3x/week (n = 109)	52 (48%)	57 (53%)	0.8
Daily (n = 20)	9 (45%)	11 (55%)	

### Nutritional changes after the cancer diagnosis

65% (n = 92) of the patients described nutritional changes after the cancer diagnosis, particularly in the consumption of fruits/ vegetables, meat and oils/ nuts (Table 4). Furthermore, 9% (n = 12) of patients implemented special diets in order to optimize the oncological treatment effect. A reduction of sugars and carbohydrates, an oil- and protein-rich diet according to Johanna Budwig as well as the Dr. Coy diet and fasting (Rudolf Breuss cancer cure) were among the most frequently used diets. Only one patient underwent formal nutritional counseling.

**Table 4:** Nutritional modifications since the cancer diagnosis

Dietary changes	Frequency (n = 92)	%
More fruits/ vegetables	61	66
Less meat	60	65
More nuts and high-quality oils	55	59
Less sweets	48	52
More fish	37	40
Less fast food	20	22

#### Fruits/vegetables

Almost none of the patients consumed the recommended five servings of fruits/ vegetables per day (Deutsche Gesellschaft für Ernährung e.V., 2017). An insufficient intake was detected in 71% (n = 99) of the patients (≤ 2 portions of fruits/ vegetables daily). However, 52% (n = 51) of these patients reported an increased intake of fruits/ vegetables after making dietary adjustments after diagnosis (Table 5).

**Table 5:** Nutrition before and after cancer diagnosis

Food	Nutrition before cancer diagnosis	Change in nutrition after cancer diagnosis
Fruit/vegetables (N = 140)		More fruit/vegetables
≥ 5 portions/day	1 (≈ 0%)	–
3–4 portions/day	40 (29%)	10 (25%)
1–2 portions/day	82 (59%)	37 (45%)
Not daily	17 (12%)	14 (82%)
Meat (N = 141)		Less meat
Daily	47 (33%)	30 (64%)
> 2 x/week	55 (39%)	19 (35%)
1–2 x/week	32 (23%)	8 (25%)
< 1 x/week	7 (5%)	3 (43%)
Fish (N = 141)		More fish
> 2 x/week	11 (8%)	3 (27%)
1–2 x/week	62 (44%)	16 (26%)
< 1 x/week	68 (48%)	18 (26%)
Convenience products (N = 140)		Less convenience products
> 2 x/week	5 (4%)	4 (80%)
1–2 x/week	13 (9%)	5 (38%)
< 1 x/week	122 (88%)	11 (9%)
Sweets (N = 139)		Less sweets
Daily	45 (32%)	24 (53%)
> 2 x/week	39 (28%)	15 (38%)
1–2 x/week	35 (25%)	7 (20%)
< 1 x/week	20 (14%)	1 (5%)

#### Consumption of meat

Assuming 150 g meat per serving, eating meat is recommended to be limited to a maximum of two times per week (Deutsche Gesellschaft für Ernährung e.V., 2017). 72% (n = 102) reported consuming more than the recommended amount of meat, and 48% (n = 49) of these patients reduced the amount of meat eaten after diagnosis. 64% of the patients (n = 30) who had eaten meat daily prior to the diagnosis of cancer reduced the intake of meat after diagnosis, demonstrating the most pronounced reduction (Table 5).

#### Fast food/sweets

There are no clear recommendations regarding the daily or weekly intake of fast food/ sweets (Deutsche Gesellschaft für Ernährung e.V., 2017). The majority of patients 88% (n = 122) reported eating fast food less than once per week. 60% (n = 84) of the patients stated that they ate sweets daily or more than twice per week. There was a more pronounced modification of the intake of sweets and fast food in patients who consumed these food groups frequently prior to the cancer diagnosis. 80% (n = 4) of the patients who consumed fast food more than twice per week (n = 5) changes their intake after the cancer diagnosis, the same was true for only 9% (n = 11) of the patients eating fast food less the once per week (n = 122). Similarly, 53% (n = 24) of the patient who ate sweets daily (n = 45) changed this after their cancer diagnosis, only 5% (n = 1) of patients who ate sweets less than once per week (n = 20) did the same (Table 5).

Table 6 shows the relation of age, BMI, use of CAM and educational degree to dietary changes following the diagnosis of cancer. Patients who implemented dietary changes were significantly younger (60.5 versus 64.8 years, *p* = 0.04, Table 6), more likely to be CAM users (*p* < 0.001, Table 6), had a lower Body-Mass-Index (*p* = 0.29, Table 6) and higher education levels (*p* = 0.11, Table 6).

**Table 6:** Dietary changes based in several variables

Charakteristics	Nutrition since cancer diagnosis		
	Improved	Unchanged	*p* value
Age ± SD	60.5 ± 11.6	64.8 ± 11.3	*p* = 0.04
BMI			*p* = 0.29
Not overweight (n = 65)	45 (69%)	20 (31%)	
Overweight (n = 71)	43 (61%)	28 (39%)	
Use of CAM			*p* < 0.001
Use of CAM (n = 84)	64 (76%)	20 (24%)	
No use of CAM (n = 57)	28 (49%)	29 (51%)	
School diploma			*p* = 0.11
None	3 (75%)	1 (25%)	
Primary school + 4 years	18 (56%)	15 (44%)	
Middle school	26 (58%)	19 (42%)	
High school diploma	45 (76%)	14 (24%)	

### Physical activity changes after the cancer diagnosis

**Fig. 2:**
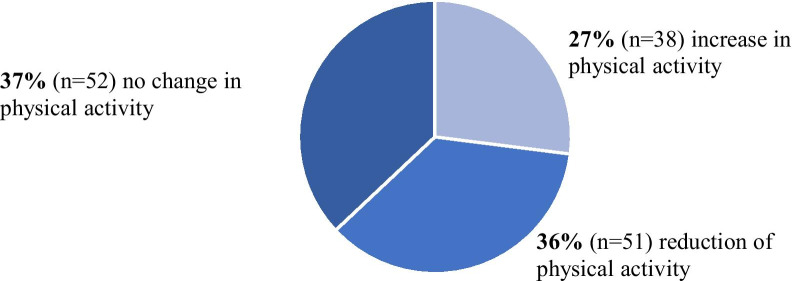
Changes in physical activity after the diagnosis of cancer

**Fig. 3:**
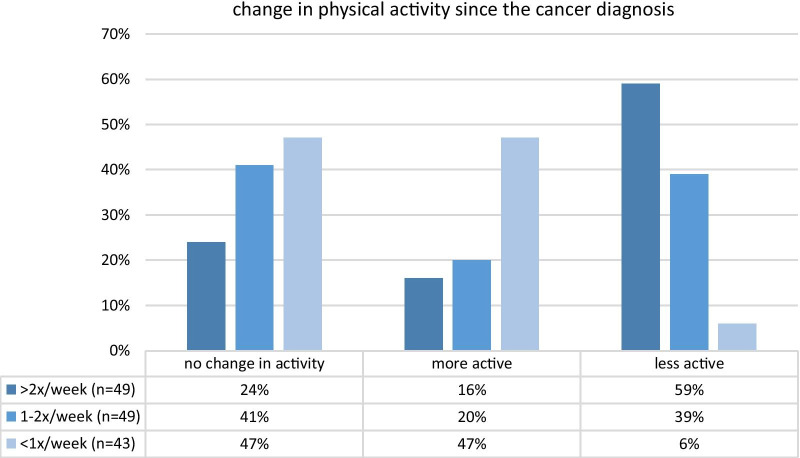
Physical activity before the cancer diagnosis and its modification after diagnosis

As shown in Fig. 2, 37% (n = 52) of the patients reported no changes in physical activity, 36% (n = 51) less exercise and 27% (n = 38) more exercise after the diagnosis of cancer. 76% (n = 39) of the patients who reduced their physical activity explained this reduction by a severe fatigue since diagnosis (“I can’t exercise a lot”, “I can’t exercise because I am physically not well enough”). Figure 3 depicts the level of physical exercise prior to the diagnosis of cancer as well as the modification in exercise thereafter. The patients who had previously exercised more than twice per week were most likely to reduce the level of their physical exercise (59%, n = 29). An increase in physical activity was reported in only 16% (n = 8) of this particular group of patients. Of the patients who had previously exercised once or twice per week, 20% (n = 10) increased their physical activity while 41% (n = 20) maintained the same level and 39% (n = 19) reported a reduction in physical exercise after diagnosis. In contrast, 47% (n = 20) of the patients who had previously exercised less than once per week increased the physical activity while 47% (n = 20) maintained the same level and 6% (n = 3) exercised less.

The relation of age, BMI, the use of CAM and the level of educational to the exercise modifications is summarized in Table 7. Patients who managed to increase their physical activity after diagnosis were significantly younger (57.4 versus 63.8 years, *p* = 0.004) and were more often cam-users (*p* = 0.009). Body-Mass-Index (*p* = 0.9) and educational level (*p* = 0.8) showed no relation to a change of physical activity.

**Table 7:** Exercise modifications based on different variables

Characteristics	Physical activity since the diagnosis of cancer		
	Increase	Unchanged/ reduced	*p* value
Age ± SD	57.4 ± 11.2	63.8 ± 11.3	*p* = 0.004
BMI			*p* = 0.9
Not overweight (n = 65)	18 (28%)	47 (72%)	
Overweight (n = 71)	19 (27%)	52 (73%)	
Use of CAM			*p* = 0.09
Use of CAM (n = 84)	27 (32%)	57 (68%)	
No use of CAM (n = 57)	11 (19%)	46 (81%)	
School diploma			*p* = 0.8
None (n = 4)	1 (25%)	1 (75%)	
Primary school + 4 years (n = 33)	7 (24%)	25 (76%)	
Middle school (n = 45)	11 (24%)	34 (76%)	
High school (n = 58)	18 (31%)	40 (69%)	

### Level of stress

This part of the questionnaire focused on the patients’ perception of stress prior to and after the diagnosis of cancer and the underlying reasons for this stress. Potential changes of the level of stress during the course of the disease and potential contributing factors were also analyzed.

Prior to the diagnosis of cancer, 41% (n = 58) of the patients reported feeling stressed on a daily basis, 26% (n = 36) stated that they often felt stressed. 24% (n = 34) claimed that they were rarely stressed, 9% (n = 13) denied feeling stressed at all.

Patients could choose between pre-written answers and a free text option in order to assess potential stressors, resulting in 17 response options that were divided into different categories. The patient population evaluated for this study described career (56%, n = 72) as well as family-associated factors (51%, n = 65) as important contributors to an increased stress level. 36% (n = 46) of the patients attributed the increased stress level to poor time management on their own part (“I make too many appointments”, “I create too much stress for myself”). 5% (n = 7) reported prior major life events as the main source of stress, and 5% (n = 5) declined to answer.

### Level of stress since the cancer diagnosis

77% (n = 108) of the patients reported a reduction in the stress level since the time of diagnosis, while 69% (n = 74) of these patients claimed to have reduced the level of stress through intentional lifestyle modifications. 14% (n = 20) noticed no changes, and 9% (n = 12) of the patients had a higher stress level after the diagnosis.

**Fig. 4:**
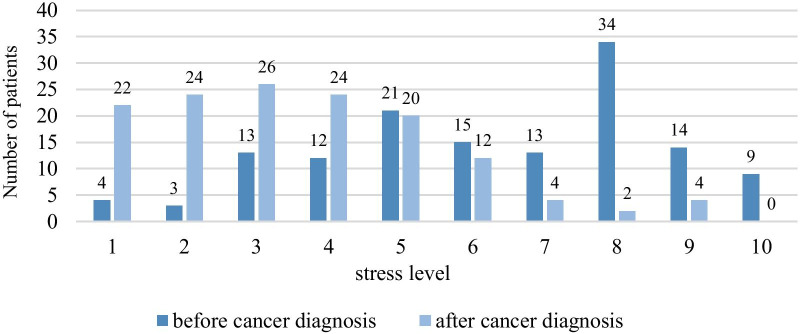
Change in stress-level since cancer-diagnosis

The level of stress based on the ordinal stress scale prior to and after diagnosis are depicted in Fig. 4 and Table 8. It shows that after the diagnosis of cancer, the level of stress diminishes in our study population. A division into low (1–3), moderate (4–7) and high (8–10) stress levels further confirms this observation (Table 8). There was a smaller proportion of patients with a high level of stress after diagnosis, underlining the reduction of the overall stress level (41% prior to, 4% after diagnosis) and an increase in the number of patients with a low stress level (14% prior to, 52% after diagnosis).

**Table 8:** Comparison of the stress level before and after the cancer diagnosis

Level of stress	Prior to cancer diagnosis (n = 138)	After cancer diagnosis (n = 138)
Low level of stress (1–3)	20 (14%)	72 (52%)
Moderate level of stress (4–7)	61 (44%)	60 (43%)
High level of stress (8–10)	57 (41%)	6 (4%)

Patients were asked to list all reasons for a change in their stress level as free text. All answers were divided into groups and are summarized in Table 9

**Table 9:** Reasons for decrease/increase in stress after diagnosis

Factors	Frequency	%
Stress reduction (n = 108)	108	77
Career-associated modifications	48	44
Mental lifestyle modifications	47	43
Family-associated modifications	23	21
Increase in stress (n = 12)	12	9
Diagnosis of cancer	8	67
Career-associated stress	2	17
Family-associated stress	2	17

A modification of the professional environment including a reduction in work hours or a leave of absence resulted in a stress reduction in the majority of patients (44%, n = 48). 43% (n = 47) attributed their lower stress level to mental lifestyle changes such as a consciously increased attentiveness to their own needs and wishes as well as the attempt to avoid mental stress due to external stressors. A reduced perception of stress due to separation/ divorce, children moving out or the death of a relative cared for by the patients was detected in 21% (n = 23) of the patients.

Patients who described an increase in stress typically attributed this stress to the underlying disease and in particular to a diminished physical capacity. 17% of the patients described a perceived increase in stress because of both family- and career-associated factors.

### Application of methods to enhance relaxation

37% (n = 53) of the patients applied relaxation techniques prior to the cancer diagnosis, particularly yoga (n = 23), autogenous training (n = 22), progressive muscle relaxation (n = 21) and meditation (n = 14). Breathing therapy, hypnosis, energy strategies, Qi Gong, Shiatsu, silent prayers and Pilates were used less frequently.

55% (n = 77) of the patients stated that they pursued some type of relaxation technique, and 39% (n = 55) of the patients were still practicing this technique at the time of the questionnaire.

37% (n = 53) of the patients had been given a recommendation to participate in relaxation techniques.

### Supportive company during the disease

Patients were questioned regarding their satisfaction with and deficits in the emotional support received during their illness.

98% (n = 138) stated that they received sufficient emotional support during their disease, particularly from friends and family (95%, n = 133). 74% (n = 103) reported feeling supported by the medical staff. Although there was a high level of satisfaction regarding the emotional support overall, 28% (n = 39) of the patients would have preferred even more intensive support, particularly from physicians (67%, n = 26) but also from family members and friends (44%, n = 17).

### Personal growth

81% (n = 114) of the patients reported a positive personal development during the course of their disease. 74% (n = 104) felt a stronger appreciation for life, 72% (n = 102) focused more on their own needs, 56% (n = 79) reported an enhanced psychological strength, 42% (n = 59) felt a higher overall satisfaction with life, and 26% (n = 37) of the patients strengthened their faith/ spirituality.

## Discussion

Lifestyle modifications in patients with gynecological malignancies were evaluated based on the nicotine and alcohol consumption, nutrition, physical exercise and the level of stress prior to and after the cancer diagnosis. The majority of patients implemented lifestyle improvements in at least one of the areas listed above after diagnosis. The implementation of positive lifestyle changes was associated with a younger age and the use of CAM.

Similar to the results of the current study, previous studies also describe a high rate of lifestyle modifications. Patterson et al. reported lifestyle modifications in 66% of breast, prostate and colorectal cancer survivors [21]. 67% of breast and 56% of cervical cancer patients described the implementation of lifestyle improvements after the diagnosis of cancer [22]. However, the comparability of these results to the current study is limited since some of the specific lifestyle factors evaluated differ between studies. Patterson et al. as well as Ashing-Giwa et al. evaluated changes in nutrition, physical activity and the use of CAM. In the current study, the use of CAM was assessed separately and was therefore not defined as a lifestyle modification. In addition, this current study also included changes in the consumption of nicotine and alcohol which might contribute to the slightly higher rate of lifestyle modifications described. Mayer et al. studied lifestyle changes (smoking, physical activity, consumption of fruits/ vegetables) using a questionnaire which was carried out with 619 cancer survivors and 2.141 participants without cancer. In contrast to the previously quoted publications, Mayer et al. did not detect a significant difference in health behavior between cancer survivors and individuals without cancer [1].

Predictors of lifestyle changes such as age have been described after a cancer diagnosis [21, 23–25]. The use of CAM seems to be associated with more active coping strategies in cancer patients [21, 26–28]. Schuerger et al. [25] described a significant difference in regular physical activity between breast and gynecological cancer patients using CAM and not using CAM (*p* = 0.007).

This suggests that CAM-users are more aware of their role and responsibility during the course of the disease and are more inclined to attempt to influence the disease by modifying their lifestyle, among other changes.

It is very likely that other psychological factors that have not been evaluated as part of this study affect the lifestyle modifications applied by cancer survivors. A functional social support system likely has a positive effect on the health behavior of cancer survivors as demonstrated by Pinto et al. in breast cancer patients who were more likely to increase their physical activity in the setting of strong social support [29].

Lutgendorf et al. [19] showed that social attachment is associated with a survival advantage for patients with ovarian cancer. The study defined two different forms of social support: “Social attachment” reflecting support through interpersonal relationships/connections and “instrumental support” meaning the availability of actual assistance. It could be shown that patients with high social attachment had a longer median survival than patients with low social attachment (4.15 years vs. 3.35 years). There was no significant association between instrumental support and survival.

These results show the importance of social support and that patient need to be screened for possible deficits in this area in order to provide support activities if necessary.

In our study current and prior smoking habits were recorded in order to assess potential changes in smoking. 63% of the patients who were smokers at the time of the questionnaire or had smoked prior stated that they reduced their nicotine consumption after the diagnosis of cancer. This corresponds to results of previous studies describing a reduction in the tobacco consumption in 46–78% of cancer survivors [30–32]. In contrast, Mayer et al. detected similar smoking habits in people with and without a diagnosed malignancy [1].

47% of the patients reported consuming less alcohol after the cancer diagnosis, specifically patients with an irregular (48% reduction) and a daily alcohol consumption (45% reduction). There was almost no association between prior drinking habits and the modifications after diagnosis. Up until now, only one study focused on modifications of the alcohol consumption in gynecologic oncology patients. This study also detected a reduction in the intake of alcohol although the consumption of alcohol was assessed by the amount of alcohol consumed (glasses/ week) and did not report the overall prevalence, making a direct comparison with the current study regarding the prevalence of the reduction of alcohol consumption impossible [33].

In the patient population evaluated for this study, 65% of women consciously altered their diet, most frequently by increasing fruits and vegetables, reducing meat, adding more nuts and high-quality oils and reducing sweets. Dietary modifications have been described in other publications following the diagnosis of cancer. Maunsell et al. described dietary modifications in 41% of breast cancer patients, Ashing-Giwa et al. in 50% of the cervical cancer patients, and Demark-Wahnefried et al. in 30–60% of cancer survivors [22, 23, 34]. Some studies also describe a reduction in fat. According to Blanchard et al., a reduction of the intake of fat was the most frequent dietary modification (51% of the patients), followed by an increase in dietary fiber (44%) and a reduction in meat (43%) [31].

In our study the modifications were more pronounced in patients whose dietary routine had been most inconsistent with official nutrition recommendations prior to the cancer diagnosis. 82% of patients who had previously not eaten fruit or vegetables on a daily basis increased the intake of these food groups while only 25% of the patients with an acceptable fruit and vegetable intake (3–4 portions per day) increased this intake further after diagnosis. Similarly, a reduction in the intake of meat, fast food and sweets were most pronounced in patients who had previously demonstrated a high consumption of these foods. These observations may indicate that the cancer diagnosis stimulated a critical assessment of the existing nutritional routine, leading to the detection and conscious improvement of deficits therein.

9% of the patients followed specific “cancer diets “ that are not evidence-based and may be associated with significant health risks [35]. Nutritional counseling might be a useful tool to inform and support cancer survivors. Only one patient received nutritional counseling which is in stark contrast to the major interest of cancer patients in health-related informative programs [25, 36].

Demark-Wahnefried et al. described that 80% of cancer survivors would have liked to have received more information on nutrition and physical activity [34]. Age, BMI, the use of CAM as well as the level of education were assessed in order to identify potential predictors for nutritional modifications and revealed a significant association between dietary changes and younger age as well as the use of CAM [21, 23, 37]. Maunsell et al. detected nutritional changes in 50% of the patients < 50 years of age, 42% of the patients aged 50–69 and 16% of the patients aged 70 and older [23].

The use of CAM and dietary modifications have not been evaluated in cancer patients to date – the current results can therefore not be compared to an existing body of literature and more research is necessary. As previously discussed, different psychological factors associated with the use of CAM may also play a role in the modification of the nutrition after a cancer diagnosis. Several studies indicate that CAM users display a more active disease coping strategy compared to non-CAM-users [27, 28].

Interestingly, 61% of overweight patients and 69% of the non-overweight patients were able to modify their diet and thereby attempt to optimize their health behavior after diagnosis of cancer. However, this also implies that 39% of the overweight patients did not change their diet. As several studies have shown that obesity can be associated with a decreased quality of life, overweight cancer patients should receive counseling regarding the benefits of a change in diet and weight reduction [38–40].

In the current study, 63% of the patients changed their physical activity after the cancer diagnosis. 36% described a reduction, 27% an increase in physical activity. The existing data regarding a modification of the exercise pattern after a cancer diagnosis are heterogeneous. Humpel et al. evaluated 657 patients diagnosed with different types of cancer and described an increase in physical activity in 31% of the participants on the study. There was a significantly higher increase in physical activity in breast and cervical cancer patients [32]. Pinto et al. detected an increase in physical activity in 42% of breast cancer patients, Ashing-Giwa et al. in 59% of breast and 50% of cervical cancer patients after the diagnosis of cancer [22, 29]. In contrast, Blanchard et al. reported an increase in physical activity in only 19% of the study participants [31]. In future studies it would be recommendable to use a uniform measurement of physical activity, for example minutes/week or metabolic equivalent of task hours/week, to allow for better comparability. Furthermore, it would be interesting to conduct of prospective longitudinal study with data collection on multiple occasions to truly understand the change in physical activity and identify possible moments for intervention.

The data of the current study demonstrate the highest reduction (59%) in physical activity after diagnosis in those cancer patients who had previously had the highest level of activity (> 2x/week). In comparison, 39% of the patients who reported exercising once to twice per week and 6% of the patients who reported exercising less than once per week were found to reduce their level of physical activity further during the course of the disease. Since oncological treatments can be associated with severe physical side effects and cancer-associated fatigue is a common symptom in up to 96% of the patients, it may be challenging to maintain the previous level of activity, possibly explaining the high exercise reduction in previously very active patients [41]. This assumption is supported by the fact that 76% of the patients who described a reduction in physical activity since diagnosis attributed this reduction to a persisting fatigue (“I am only able to exercise less”, “I cannot exercise at all because I feel so unwell”).

Patients with a very low level of activity (less than once per week) prior to the diagnosis reported the biggest increase in physical activity. This result is consistent with the observations on nutritional modifications where positive alterations were most pronounced in patients who had previously followed a diet that was very discrepant to official nutritional recommendations.

In order to identify potential predictors, the relation of several variables on the modification of physical activity was evaluated (age, body-mass-index, use of complementary medicine, education) and revealed that younger patients were significantly more likely increase their physical activity after the cancer diagnosis. Patterson et al. examined the influence of age, education and cancer-type on the implementation of physical activity after cancer diagnosis and found no significant correlations [21]. Similarly, studies by Humpel et al. and Blanchard et al. found no association between age/education and an increase in physical activity [31, 32]. Contrary to the findings, the HEAL (Health, Eating, Activity and Lifestyle) study examined 856 breast cancer patients and revealed a significantly higher decrease in physical activity in association with higher age and obesity [42]. Due to these inconsistent results, it is currently not possible to give a concrete statement about the influence of different variables on the change of physical activity showing that further studies are needed to reveal possible associations.

The average level of stress prior to diagnosis and at the time of the questionnaire was used to assess a potential stress reduction. An ordinal scale (1–10) was used for the classification of the level of stress, with increasing numbers indicating an increase in stress. The use of this ordinal scale allowed a direct comparison between the level of stress prior to and after diagnosis, revealing a stress reduction in 77% of the patients. The most pronounced differences were detected between the categories “low stress level (1–3) “and “high stress level (8–10)”. 41% of the patients described a high stress level prior to diagnosis while only 4% reported a high stress level at the time of the questionnaire. The category”low stress level “ revealed the contrary (an increase in stress from 14 to 52%). These results suggest that the diagnosis of a gynecological malignancy may initiate a reconsideration of the current life situation and can lead to the implementation of active stress reduction mechanisms. Consistent with the current study, a stress reduction has also been described by other studies after a cancer diagnosis [43]. Wang et al. evaluated 235 breast cancer patients regarding different lifestyle modifications prior to and after diagnosis and detected a significant reduction in perceived stress after the cancer diagnosis [44].

Other studies, however, report a higher level of stress and a higher incidence of depression in cancer survivors [15–17]. These studies did not assess the stress level prior to diagnosis and can therefore not comment on the changes in stress during the disease. Aside from a potential stress reduction, 81% of patients describe undergoing a positive development because of the cancer diagnosis. A higher appreciation for life, more insight into the own needs, a more profound faith and greater psychological strength as well as a higher satisfaction are among the positive changes described. This common phenomenon in cancer survivors is referred to as “post-traumatic growth” and “benefit finding” in the literature. Hodgkinson et al. [45] reported a subjective benefit in 68% of cancer survivors of gynecological malignancies.

## Conclusion

In the current study population, various positive lifestyle changes were detected after the diagnosis of cancer, but many patients were unable to fully exhaust the potential for improvement. As many studies point towards a beneficial effect of lifestyle modifications on prognosis and quality of life of cancer patients it is important to add further information on the implementation of such lifestyle changes to better understand the patients needs and to integrate relevant aspects into oncological treatment plans.

### Strengths and limitations

The strengths of the study are the fact that the data were obtained in a very homogenous population of women diagnosed with gynaecological malignancies and treated at a single gynaecological cancer center. The recommendations regarding lifestyle modifications were provided by the same provider. The patient population included in this study was therefore treated according to the same treatment standards.

This study has several limitations.

First, the presented data was obtained via telephone interview. The goal of the telephone interview was to clear up any misunderstandings or questions in order to obtain a complete and accurate data set. However, it has to be taken into account that a personal interview might lead participants to answer not entirely honestly if they suspect the researcher to have a preference.

Secondly, as this study is of a retrospective nature it is possible that patients do not remember all details thus leading to inaccurate data via recall bias.

Thirdly, we cannot rule out that non-respondents have the same behavior as respondents. It is possible that patients with less favorable health behavior prior to the cancer diagnosis and/or patients that did not change their health behavior might be less inclined to participate in the study. Therefore, the presented data regarding changes in health behavior might be inflated. Further, there was no confirmatory hypothesis testing and respective power calculations for sample size estimation. All results are of exploratory nature.

## Supplementary Information

**Additional file 1.** Questionnaire.

## Data Availability

The datasets used and/or analysed during the current study are available from the corresponding author on reasonable request.

## Abbreviations

BMI: Body mass index
CAM: Complementary and alternative medicine
SD: Standard deviation
