# Supplementary material for: Lifestyle modifications after the diagnosis of gynecological cancer
Source: BMC Womens Health. 2021 Jun 28;21:260. doi: 10.1186/s12905-021-01391-5 (PMC8240378; doi:10.1186/s12905-021-01391-5)
Supplement: Supplementary file 1 — Additional file 1. Questionnaire. [file 12905_2021_1391_MOESM1_ESM.docx]

**Part 1: Clinical and sociodemographic data**

***Regarding cancer, do you use complementary/alternative medicine (CAM)?***

O yes

O no

***Do you know of the consultation-hour for CAM at the university hospital Rechts der Isar?***

O yes

O no

***What type of CAM did you use?***

O trace element preparations, O vitamins, O phytotherapy, O homeopathy, O anthroposophical medicine, O dietary supplements, O TCM (traditional chinese medicin), O medicinal tea , O infusions to strengthen the immune system O energetic healing , O detox, O hyperthermia, O schüßler salts, O medicinal mushrooms, O enzyme therapy, O other

***What type of gynecological cancer do you/did you suffer from?***

O ovarian cancer

O cervical cancer

O endometrial cancer

***When were you diagnosed with cancer?***

_________________

***Was there a cancer recurrence?***

O yes

O no

O unknown

***Were metastasis found?***

O yes

O no

O unknown

***Did you receive chemotherapy?***

O yes

O no (not necessary)

O no (refusal)

***Did you receive radiation?***

O yes

O no (not necessary)

O no (refusal)

***Did you receive hormone therapy?***

O yes

O no (not necessary)

O no (refusal)

***Did you receive antibody therapy?***

O yes

O no (not necessary)

O no (refusal)

***How old are you?***

_______

***How old were you when the cancer was diagnosed?***

_______

***How tall are you?***

_______

***How much do you weigh?***

________

***What is your family status?***

O married

O relationship

O single

O divorced

O widowed

***Did your family status change due to cancer?***

O yes

O no

***What educational degree do you have?***

O no educational degree

O Primary school ±4 years

O Middle school diploma

O High school diploma

***What kind of work did you do up to the point of diagnosis?***

O unemployed

O employee

O self-employed

O official

***Did you choose early retirement due to your cancer diagnosis?***

O yes

O no

**Do you feel as energetic as before your diagnosis?**

O yes

O no

***Are you religious/ spiritual?***

O yes

O no

O no comment

**Part 2: Use of CAM**

***Did you use CAM before being diagnosed with cancer?***

O yes

O no

***If so, when did you use it?***

O common cold, O gastrointestinal disorders, O childhood diseases, O menstruational pain, O skin diseases, O allergies, O bladder problems, O nervousness, O injuries, O cancer, O strengthening of the immune system, O menopausal problems, O pain, O musculoskeletal problems, O loss of hair, O cardiac disease, O exhaustion, O thyroid problems, O dizziness, O tinnitus, O migraine

***Regarding your illness, were you recommended the use of complementary therapies?***

O yes

O no

***If so, by who?***

O gynecological hospital Rechts der Isar, O consultation for complementary medicine, O family doctor, O internist, O gynecologist, O radiotherapist, O oncologist, O naturopathic doctor, O friends, O family, O self-help-group, O health insurance, O apothecary, O neurologist, O psychotherapist, O alternative practitioner, O osteopath, O rehabilitation clinic, O other patients, O orthopedist, O physiotherapist, O immunologist

***What therapies were recommended?***

O trace element preparations, O vitamins, O phytotherapy, O homeopathy, O anthroposophical medicine, O dietary supplements, O TCM (traditional chinese medicin), O medicinal tea , O infusions to strengthen the immune system O energetic healing , O detox, O hyperthermia, O schüßler salts, O medicinal mushrooms, O enzyme therapy, O other

***Did you follow this recommendation?***

O yes

O no

***If not, would you have liked to receive information by your doctors regarding the use of CAM?***

O yes

O no

O unknown

***Did someone advise against the use of CAM regarding your illness?***

O yes

O no

***If so, who advised against the use of CAM?***

O gynecological hospital Rechts der Isar, O consultation for complementary medicine, O family doctor, O internist, O gynecologist, O radiotherapist, O oncologist, O naturopathic doctor, O friends, O family, O self-help-group, O health insurance, O apothecary, O neurologist, O psychotherapist, O alternative practitioner, O osteopath, O rehabilitation clinic, O other patients, O orthopedist, O physiotherapist, O immunologist

***Which CAM-therapies were advised against?***

O trace element preparations, O vitamins, O phytotherapy, O homeopathy, O anthroposophical medicine, O dietary supplements, O TCM (traditional chinese medicin), O medicinal tea , O infusions to strengthen the immune system O energetic healing , O detox, O hyperthermia, O schüßler salts, O medicinal mushrooms, O enzyme therapy, O other

***Did you follow this recommendation?***

O yes

O no

***Where did you research/gather information regarding CAM?***

O I did not research CAM therapies, O doctor, O self-help group, O german cancer society, O CAM-brochure, O medical journal/book, O internet, O TV, O alternative practitioner, O health insurance, O apothecary, O friends, O family, O lecture about CAM, O I looked for a doctor/clinic who provides CAM, O I looked for an alternative practitioner, O CAM consultation, O other patients, O cancer information service O patient-information day at university hospital Rechts der Isar

***Which therapies did you research?***

O trace element preparations, O vitamins, O phytotherapy, O homeopathy, O anthroposophical medicine, O dietary supplements, O TCM (traditional chinese medicin), O medicinal tea , O infusions to strengthen the immune system O energetic healing , O detox, O hyperthermia, O schüßler salts, O medicinal mushrooms, O enzyme therapy, O other

***Why did you decide to use CAM-therapies?***

O recommendation/positive reports, O active involvement in recovery, O wish for holistic medicine, O use of all potential treatments, O convinced in CAM therapies, O prevention and treatment of side effects of the conventional therapy, O information about CAM through books/radio/TV/lectures, O I feel like I receive better care by doctors who provide CAM, O loss of faith in conventional medicine, O conventional medicine did not work

***What were the reasons for not using CAM-therapies?***

O I was not informed about CAM, O I felt that the conventional therapy was sufficient, O I did not believe that CAM therapies would work, O fear of fraud, O fear of interactions with conventional treatments, O fear of side effects, O too expensive

***Which CAM-therapies do you use mostly?***

O trace element preparations, O vitamins, O phytotherapy, O homeopathy, O anthroposophical medicine, O dietary supplements, O TCM (traditional chinese medicin), O medicinal tea , O infusions to strengthen the immune system O energetic healing , O detox, O hyperthermia, O schüßler salts, O medicinal mushrooms, O enzyme therapy, O other

***Would you use CAM-therapies if your illness progressed?***

O yes

O no

***How do you use CAM-therapies?***

O additive/complementary to conventional therapies

O alternative/instead of conventional therapie

***When did you start using CAM therapies?***

O when I was first diagnosed, O during chemotherapy/radiotherapy, O after surgery, O after I had completed the conventional treatment, O after progression of my illness (recurrence of cancer/metastasis)

***How often do you use this CAM therapies?***

O daily, O 1-3x/weeks, O in blocks, O irregular

***Do you use CAM therapies as often as it was recommended?***

O yes

O no

O I didn’t receive a recommendation

***If so, why did you use it less often?***

O the cost, O expenditure of time, O forgetfulness, O I do not believe in its effectiveness, O I don’t feel an effect of the CAM therapy, O I do not want to take so many medications

***Compared to conventional treatments, how important are CAM therapies to you?***

O more important, O as important, O less important

***What hopes are related to your CAM-therapies?***

O healing (fight cancer, protection from metastasis/recurrence of cancer, strengthen self-healing), O quality of life (more vitality, stable psyche, less pain), O strengthen the immune system, O support of the conventional treatment, O reduce side effects of the conventional treatment, O replacement of conventional treatment

***Has your quality of life improved by using CAM?***

O yes

O no

***If so, how?***

O physical improvement (more vitality, less side effects of conventional treatment, less pain)

O improvement of psyche (more hope, more stable psyche)

***Which therapies did you find especially helpful?***

O trace element preparations, O vitamins, O phytotherapy, O homeopathy, O anthroposophical medicine, O dietary supplements, O TCM (traditional chinese medicin), O medicinal tea , O infusions to strengthen the immune system O energetic healing , O detox, O hyperthermia, O schüßler salts, O medicinal mushrooms, O enzyme therapy, O other

***How much money do you spent per month on CAM therapies?***

O 1-50€, O >50-100€, O >100€

***Are you reimbursed by your health insurance?***

O yes, completely, O partially, O no, O unknown

***Are you privately insured?***

O yes

O no

***Would more financial support move you to change your health insurance?***

O yes

O no

***Would you recommend the use of CAM therapies to other patients?***

O yes

O no

***Would you wish for more doctors tob e educated/knowledgeable in the field of CAM?***

O yes

O no

O unknown

***Would you wish for CAM tob e fully integrated in the health care system?***

O yes

O no

O unknown

**Part 3: Change of lifestyle factors after cancer diagnosis**

***How would you describe your lifestyle before your cancer diagnosis?***

O healthy

O not healthy

O average

***Did you change your lifestyle after cancer diagnosis?***

O healthier

O less healthy

O unchanged

***Do you smoke?***

O yes

O no

***If not, did you smoke within the last 5 years?***

O yes

O no

***Did you reduce your tabacco consumption after cancer diagnosis?***

O yes

O no

***Did you drink alcohol before cancer diagnosis?***

O daily

O up to 3x/week

O no

***Did you reduce your alcohol consumption after cancer diagnosis?***

O yes

O no

***Did you have a balanced diet before cancer diagnosis?***

O yes

O no

***How many times/portions per week did you consume:***

***fish?***

O >2 x/week, O 1-2 x/week, O <1 x/week

***Meat?***

O daily, O >2 x/week, O 1-2 x/week, O <1 x/week

***Convenience products?***

O >2 x/week, O 1-2 x/week, O <1 x/week

***sweets?***

O daily, O >2 x/week, O 1-2 x/week, O <1 x/week

***Fruits/vegetables?***

O ≥5 portions/day, O 3-4 portions/day, O 1-2 portions/day, O not daily

***Did you change your nutrition after cancer diagnosis?***

O yes

O no

***I made the following changes:***

O more fruit/vegetables

O less meat

O more nuts

O less sweets

O more fish

O less convenience products

***Did you follow a special diet, if so which?***

__________________________________________________________________________________

***How many times per week did you do pyhsical activity?***

O <1 x/week, O 1-2 x/week, O >2 x/week

***Have you changed the amount of physical activity after cancer diagnosis?***

O increased

O reduced

O no change

***Before cancer diagnosis, did you often feel stressed?***

O yes

O no

***Why were did you feel stressed?***

__________________________________________________________________________________

***On a scale from 1-10, how high would you say your level of stress was before cancer diagnosis?***

_______

***Did your level of stress change after cancer diagnosis?***

O I feel more stressed

O I feel less stressed

O unchanged

***Why did your level of stress change after cancer diagnosis?***

__________________________________________________________________________________

***On a scale from 1-10, how high is your current level of stress?***

________

***Did your level of stress change due to conscious lifestyle changes?***

O yes

O no

***Did you use relaxation techniques (yoga, meditation etc.) before cancer diagnosis?***

O yes

O no

***If so, what relaxation technique did you use?***

__________________________________________________________________________________

***Did you use relaxation techniques after cancer diagnosis?***

O yes

O no

***Was the use of relaxation techniques recommended to you after cancer diagnosis?***

O yes

O no

***Did you receive psychooncological counseling?***

O yes

O no

***Did you partake in rehabilitation?***

O yes

O no

***Did you feel like you had enough support during the time of you illness***

O yes

O no

***Who supported you the most***

O doctors, O family, O friends

***I would have liked more support by:***

O doctors, O family, O friends, O not applicable

***What gave you strength during your illness?***

__________________________________________________________________________________

***Do you believe that your illness might have led to a positive change in your life?***

O I lead a more conscious life

O I pay more attention to my own needs

O I am more satisfied with my life

O I am not as easily stressed

O My illness has made me stronger

O My illness has strengthened my faith

O other:___________________________________________________________________________

O no
